# Effect of Filler Types on Cellulose-Acetate-Based Composite Used as Coatings for Biodegradable Magnesium Implants for Trauma

**DOI:** 10.3390/ma16020554

**Published:** 2023-01-06

**Authors:** Alexandru Streza, Aurora Antoniac, Veronica Manescu (Paltanea), Gheorghe Paltanea, Alina Robu, Horatiu Dura, Liliana Verestiuc, Enache Stanica, Stefan Ioan Voicu, Iulian Antoniac, Mihai Bogdan Cristea, Bogdan Radu Dragomir, Julietta V. Rau, Maria-Magdalena Manolea

**Affiliations:** 1 Faculty of Material Science and Engineering, University Politehnica of Bucharest, 313 Splaiul Independentei Street, District 6, 060042 Bucharest, Romania; 2 Faculty of Electrical Engineering, University Politehnica of Bucharest, 313 Splaiul Independentei Street, District 6, 060042 Bucharest, Romania; 3 Faculty of Medicine, Lucian Blaga University of Sibiu, 10 Victoriei Boulevard, 550024 Sibiu, Romania; 4 Faculty of Medical Bioengineering, University of Medicine and Pharmacy “Grigore T. Popa”, 16 University Street, 700115 Iasi, Romania; 5 National Institute for Cryogenics and Isotopic Technologies ICSI-Rm. Valcea, ICSI Energy, 4 Uzinei Street, 240050 Râmnicu Vâlcea, Romania; 6 Faculty of Applied Chemistry and Materials Science, University Politehnica of Bucharest, 1-7 Gheorghe Polizu Street, District 1, 011061 Bucharest, Romania; 7 Academy of Romanian Scientists, 54 Splaiul Independentei Street, District 5, 050094 Bucharest, Romania; 8 Department of Morphological Sciences, “Carol Davila” University of Medicine and Pharmacy, 37 Dionisie Lupu Street, 020021 Bucharest, Romania; 9 Faculty of Dental Medicine, University of Medicine and Pharmacy “Grigore T. Popa”, 16 University Street, 700115 Iasi, Romania; 10 DDD Medical Services SRL, 78 Vasile Lupu Street, 700350 Iasi, Romania; 11 Istituto di Struttura della Materia, Consiglio Nazionale delle Ricerche (ISM-CNR), Via del Fosso del Cavaliere 100, 00133 Rome, Italy; 12 Department of Analytical, Physical and Colloid Chemistry, Institute of Pharmacy, I.M. Sechenov First Moscow State Medical University, 8 Trubetskaya Street, Build. 2, 119991 Moscow, Russia; 13 Department of Obstetrics and Gynecology, University of Medicine and Pharmacy of Craiova, 2 Petru Rares Street, 200349 Craiova, Romania

**Keywords:** magnesium coatings, magnesium alloys, cellulose composite

## Abstract

Magnesium alloys are considered one of the most promising materials for biodegradable trauma implants because they promote bone healing and exhibit adequate mechanical strength during their biodegradation in relation to the bone healing process. Surface modification of biodegradable magnesium alloys is an important research field that is analyzed in many publications as the biodegradation due to the corrosion process and the interface with human tissue is improved. The aim of the current preliminary study is to develop a polymeric-based composite coating on biodegradable magnesium alloys by the solvent evaporation method to reduce the biodegradation rate much more than in the case of simple polymeric coatings by involving some bioactive filler in the form of particles consisting of hydroxyapatite and magnesium. Various techniques such as SEM coupled with EDS, FTIR, and RAMAN spectroscopy, and contact angle were used for the structural and morphological characterization of the coatings. In addition, thermogravimetric analysis (TGA) was used to study the effect of filler particles on polymer thermostability. In vitro cytotoxicity assays were performed on MG-63 cells (human osteosarcomas). The experimental analysis highlights the positive effect of magnesium and hydroxyapatite particles as filler for cellulose acetate when they are used alone from biocompatibility and surface analysis points of view, and it is not recommended to use both types of particles (hydroxyapatite and magnesium) as hybrid filling. In future studies focused on implantation testing, we will use only CA-based composite coatings with one filler on magnesium alloys because these composite coatings have shown better results from the in vitro testing point of view for future potential orthopedic biodegradable implants for trauma.

## 1. Introduction

**Figure 1:**
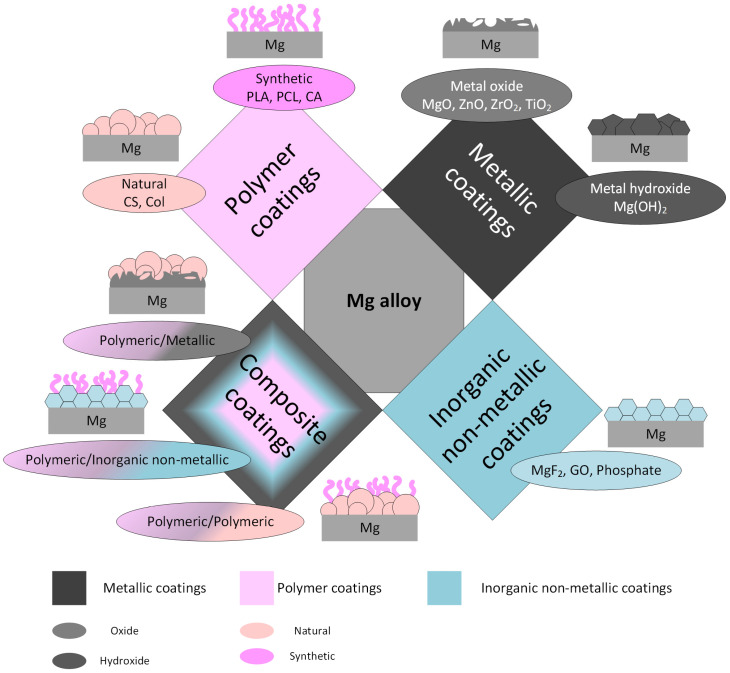
Main types of coatings for Mg-based alloys.

Magnesium alloys are considered one of the most promising biodegradable materials due to their outstanding mechanical strength and lightweight properties. They are appropriate for biodegradable implant manufacture because they promote bone healing and osteointegration and exhibit adequate mechanical strength at the beginning of the implantation process. After some time, due to physiological media action, they are progressively degraded [1,2,3]. Surface modification of Mg-based alloys is an important research field that is analyzed in many publications due to the fact that corrosion resistance, biocompatibility, and mechanical properties are improved [4,5,6,7]. The most used techniques are mechanical methods (abrasion, friction, and shot peening) [8], ion implantation [9], chemical conversion, sol–gel, micro-arc oxidation (MAO), layer-by-layer self-assembly, and alloy coating. The material coating is considered an adequate method to improve the degradation rate of Mg-based alloys, being highly biocompatible and corrosion-resistant [10,11]. Further, we use the classification of coatings from the material point of view. The literature reports different types such as metallic (metal oxide and metal hydroxide), inorganic non-metallic (MgF_2_, phosphates, and graphene oxide), polymeric (synthetic and natural), and composite coatings (Figure 1).

The main metal coatings are based on the formation of oxides and hydroxides of the respective metals. They act as an inert phase, which protects the Mg-based alloys, increasing the corrosion resistance. The oxide coatings consist of magnesium, zirconium, and titanium oxide. Pan et al. [12] studied different oxide coatings, such as zinc oxide (ZnO) and magnesium oxide (MgO), which were applied to Mg-6Zn-0.6Zr (ZK60) alloy, and reported increased bioactivity, decreased corrosion phenomenon, and a high osteoblast adhesion rate. Yang et al. [13] prepared a dense zirconium oxide (ZrO_2_) nanocoating on the surface of Mg-Sr alloy using atomic layer deposition. Corrosion tests showed that the ZrO_2_ film played an important role in decreasing corrosion rates. Hou et al. [14] developed a 400 nm thickness titanium dioxide (TiO_2_) coating based on the magnetron sputtering method on the Mg-Zn alloy surface. Due to the amorphous and dense coating, the corrosion behavior and biocompatibility were highly improved. Peron et al. [15] made thin TiO_2_ coatings deposited on Mg-3Al-1Zn (AZ31) through sputtering or atomic layer deposition. The coated samples exhibited an important reduction in the corrosion current density and hydrogen amount.

A dense magnesium hydroxide Mg(OH)_2_ film can be prepared based on hydrothermal treatment on the surface of Mg alloys to decrease their corrosion rate. By adjusting the hydrothermal treatment time, the thickness of the protective film can be controlled. Feng et al. [16] developed a Mg(OH)_2_ coating on Mg-9Al-1Zn (AZ91) alloy. It was concluded that the hydrothermal treatment time and the pH value significantly impact the morphology and corrosion resistance. Xu et al. [17] proposed a similar analysis consisting of a Mg(OH)_2_ coating obtained based on one-step hydrothermal treatment on Mg-6Zn-0.6Zr (ZK60) alloy. The best morphology and corrosion resistance of the Mg(OH)_2_ coating were observed after 24 h of hydrothermal treatment and at 120 °C.

Inorganic non-metallic coatings are another important class of surface modification applied to Mg-based alloys. They consist of magnesium fluoride (MgF_2_), graphene oxide (GO), and phosphate coatings. A MgF_2_ coating is obtained after treatment with hydrofluoric acid. The fluorine conversion layer exhibits a compact structure extensively used in the surface coatings of biomedical Mg-based implants. Drynda et al. [18] investigated Mg-Ca alloys coated with fluoride. The corrosion resistance of the material was improved, and no adverse reactions, such as inflammation or hyperplasia, were reported. Chiu et al. [19] developed an MgF_2_ coating through a conversion treatment that was analyzed by the chemical impedance spectroscopy method, and it proved to increase the corrosion resistance by an average of 35 times.

Graphene oxide (GO) has a unique two-dimensional structure with high surface energy and functional groups such as carboxyl, hydroxyl, and epoxy. GO is characterized by good hydrophilicity and dispersibility. Fernandez et al. [20] prepared two types of reduced graphene oxide on Mg alloys using chemical and electrochemical methods. It was noticed that the GO-coated samples exhibited a reduction of 80% in the corrosion rate. Because GO has poor wettability with a metal matrix and weak interfacial bonding strength, it is usually used in composite coatings based on the layer-by-layer self-assembly method.

Another important type of inorganic non-metallic coating is phosphate coating. In the literature, it was shown that different ceramic-coated implants exhibited high osseointegration properties. Calcium phosphates coatings improved the biocompatibility, corrosion resistance, and osteoconductivity of Mg-based implants. Mahapatro et al. [21] showed that hydroxyapatite (HAp) promoted cell adhesion and proliferation but exhibited low mechanical properties, which makes this material unsuitable for load-bearing applications. Song et al. [22] prepared fluoridated hydroxyapatite that induced a low solubility in physiological media and increased biocompatibility when applied to an Mg-Zn alloy. Seyfoori et al. [23] deposited phosphate film and silicate film coatings separately on the surface of Mg-3Al-1Zn (AZ31) alloy using the micro-arc oxidation technique. Regarding the phosphate film, it was noticed that it favored a lower weight loss of material, and a reduced pH, compared with the silicate coating. Supplementary to these samples, a higher proliferation of the osteosarcoma cells was put in evidence. This fact was due to the higher roughness and corrosion resistance of the surface coating with phosphate film.

Polymer coatings comprise synthetic or natural polymers. They exhibit multifunctional properties that can enhance biocompatibility and increase the corrosion resistance of Mg-based implants. The degradable polymer coating can be used in the theragnostic domain to deliver different drugs. Biodegradable polymers slowly degrade due to the chemical reactions that take place, classified into two main groups, oxidation and hydrolysis, which can occur either simultaneously or successively [24,25,26,27,28]. Table 1 summarizes the most used synthetic polymers as coatings for Mg-based alloys, such as polylactic acid (PLA) [29,30], poly(Lactide-Co-Glycolic) acid (PLGA) [31], polycaprolactone (PCL) [32,33], and cellulose acetate [26,34].

Various groups have proposed natural polymers such as chitosan [35], collagen [36], gelatin, silk fibroin [37,38,39], alginate [40], and hyaluronic acid [41] for coating biodegradable Mg-based alloys. Gelatin natural polymer can be part of a composite complex, formed with other polymers [42,43], or with other materials [44].

**Figure 2:**
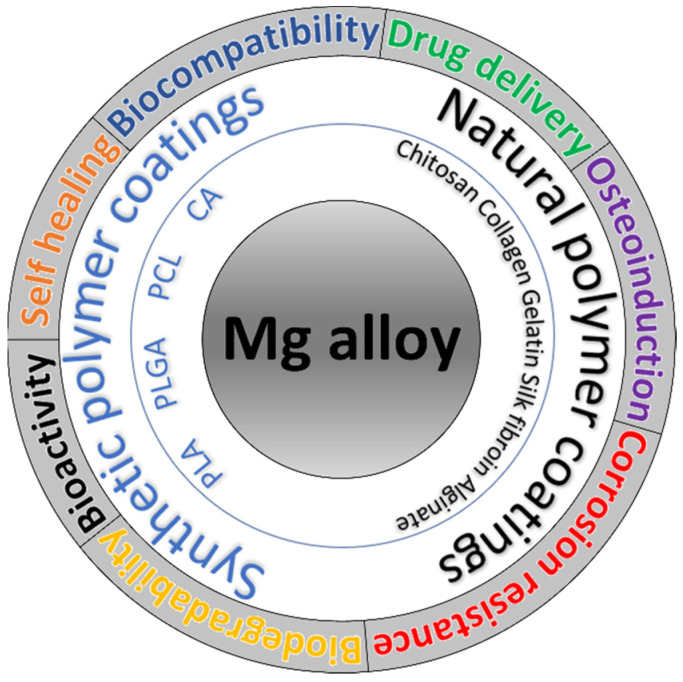
The main synthetic and natural polymer coatings on Mg-based alloys and their main functionalities in the medical domain.

Figure 2 presents the most important medical functionalities of the Mg-based implants with polymer coatings.

The composite coatings are characterized, in general, by better corrosion resistance and increased biocompatibility [42,45,46,47,48,49]. Some examples of composite coatings based on polymers applied in the case of Mg-based alloys are presented in Table 2. Although many studies are reported in the literature regarding this topic, the strength of the interfacial bonding force among the coating layers and the complex degradation process must be further investigated. The composite coatings can have great potential in the case of Mg alloys, and much research must be conducted regarding in vivo experiments to evaluate and compare the biological activity of such implants.

The aim of the current study is to develop a polymeric-based composite coating on biodegradable magnesium alloys to reduce the biodegradation rate much more than in the case of simple polymeric coatings by involving some bioactive filler in the form of particles consisting of hydroxyapatite and magnesium. Composite coatings for osseointegration are designed to be used at the interface between implants and bone to facilitate and improve implant integration in bone.

## 2. Materials and Methods

Cellulose acetate (Sigma-Aldrich, St. Louis, MO, USA) was dissolved in dimethylformamide (Sigma-Aldrich, St. Louis, MO, USA) at a concentration of 12 wt%. The hydroxyapatite (HAp) and magnesium (Mg) particles were dispersed in the polymer solution by ultrasonication for 30 min. to obtain a homogeneous solution. The particles were added relative to the amount of pure cellulose acetate polymer from the solution, according to Table 3. The samples were obtained by casting the mixtures into a Petri vessel with a diameter of 10 cm and evaporating the solvent at 50 °C for 24–48 h in an oven.

Finally, the samples were washed with distilled water and ethanol and kept dry until their investigation.

### 2.1. The Samples’ Surface Morphology and Elemental Composition

The Samples’ Surface Morphology and Elemental Composition were examined using a QUANTA INSPECT F Scanning Electron Microscope (FEI Company, Eindhoven, The Netherlands) coupled with an Energy-Dispersive X-Ray Spectrometer Detector (EDAX).

### 2.2. Infrared Spectroscopy

Infrared Spectroscopy was performed on a JASCO FTIR 6200 spectrometer (JASCO International Co., Tokyo, Japan) using an ATR device in the spectral range of 600–4000 cm^−1^.

### 2.3. Raman Spectroscopy

Raman Spectroscopy was carried out by using an alpha 300 RAS+ system (WITEC, Ulm, Germany) equipped with a 532 nm laser (75 mW) in conjunction with a grating spectrometer (600 grooves/mm) and a microscope (50× magnification). For spectra acquisition, the integration time was 2 s per spectrum. To enhance the signal-to-noise ratio, the spectra were averaged over 20 successive measurements. Due to the optical system alignment and the grating dispersion with respect to the incident laser line, the Raman spectra shifted with up to 8 cm^−1^ over the spectral range between 200 and 3800 cm^−1^.

### 2.4. The Contact Angle Measurements

The Contact Angle Measurements were made using A KRÜSS DSA30 Drop Shape Analysis System to determine the wettability properties of the sample’s surface. The obtained images were processed by aligning the tangent at the profile of the sessile drop at the point of contact with the surface. Contact angle measurements were made in triplicate, and an average value was calculated.

### 2.5. Thermogravimetric (TGA) Curves

Thermogravimetric (TGA) Curves were simultaneously obtained using a TA Instruments SDT Q600 system with a constant heating rate of 10 °C per minute in a nitrogen working atmosphere over the temperature range of 10–600 °C.

### 2.6. Degradation and Swelling Studies

Degradation and Swelling Studies were performed using phosphate-buffered saline solution (PBS, pH = 7.4) under conditions that simulate the human body. Phosphate-buffered saline solution (PBS) was obtained in the laboratory by mixing the following reagents, sodium chloride (NaCl), potassium chloride (KCl), potassium phosphate monobasic (KH_2_PO_4_), disodium hydrogen phosphate (Na_2_HPO_4_), and deionized water. All reagent-grade chemicals were purchased from the Sigma-Aldrich (St. Louis, MO, USA) company.

The swelling was evaluated using the gravimetric method. Each type of sample was weighed (*W_i_*) and then immersed in 10 mL of phosphate-buffered saline solution, at 37 °C, for different periods (15, 30, 45, 60, 90, 120, 1440, 2880, and 4320 min). For each period, after removing the sample from the medium, they were lightly dried on filter paper and weighed (*W_f_*). The swelling rate was calculated as follows:

(1) $$S.R.\;(\%)=\frac{W_{i}-W_{f}}{W_{f}}\times 100$$

The degradation study was performed by immersing the samples in a PBS medium at 37 °C for 5 weeks. After immersion, the samples were dried in a desiccator to a constant mass and reweighed. The degradation degree was evaluated by determining the weight loss of the experimental samples at 1, 2, 3, 4, and 5 weeks of immersion as follows:

(2) $$W.L.\;(\%)=\frac{W_{i}-W_{f}}{W_{i}}\times 100,$$

where: *W_i_* is the initial mass value before immersion; *W_f_* is the final mass value after the sample has reached constant mass.

In the case of both studies, the medium was changed every day, and each data point represents the average of three individual measurements.

### 2.7. In Vitro Cytotoxicity Assays

All reagents/kits used for cell culture were obtained from Sigma-Aldrich (Steinheim, Germany), unless otherwise indicated. MG-63 cells (human osteosarcomas) were seeded in plates of 48 wells at a density of 12 × 10^3^ cells/well for 24 h followed by the addition of the samples on top of the cell layer and incubation.

All tested materials were sterilized by exposure to UV radiation for 2 h (1 h on each side). Two methods were used to evaluate in vitro cytotoxicity:

- Indirect contact method for particle materials (hydroxyapatite—HAp and magnesium particles—Mg) used to obtain the composite materials. The extracts were prepared by immersion of 2 mg/mL of each particle material in Dulbecco’s modified Eagle’s medium (DMEM) with 1% P/S/N, under stirring (200 rpm, 37 °C) for 24 h and filtering (70 μm), and finlaly mixed with 10% FBS.
- Direct contact method for CA, CA-HAp, CA-Mg, and CA-HAp-Mg samples, after materials’ incubation with DMEM, suplemented with 10% FBS and 1% P/S/N.

### 2.8. MTT Assay

To perform the test, MTT solution (5% in DMEM) was incubated with cells at 37 °C for 2 h 30 min and the resulting formazan [50] was solubilized with DMSO (500 μL/well). The absorbance of the resulting formazan solution was measured at a wavelength of 570 nm (plate reader Tecan Sun-rise Plate Reader) and compared to the control. The calculated ratio represented the cell viability:

(3) $$\mathrm{Cell}\;\mathrm{viability}\;(\%)=\frac{abs\;extract}{abs\;control}\times 100,$$

where *abs extract* represents the absorbance of the extract, while *abs control* is the absorbance of the control.

The MTT test was performed for both contact methods at 24 and 72 h, respectively, in triplicate. To sustain the MTT results, cell morphology and density were studied with an Inverted Phase-Contrast Microscope (Leica, Wetzlar, Germany) by taking representative images (10× objective).

The MTT test results were analyzed using two-way ANOVA by means. All statistical analyses were performed using Tukey’s post hoc analysis.

The Calcein-AM cell viability assay was carried out at 72 h. The cells were washed twice with HBSS (with calcium and magnesium, without phenol red) and then incubated with Calcein solution (2 μL of calcein to 1 mL of HBSS with calcium and magnesium) for 40 min at 5.5% CO_2_, 37 °C, and 95% relative humidity and, finally, the samples were imaged with an inverted microscope with a phase contrast system and fluorescence (Leica, Wetzlar, Germany) to analyze cell morphology.

## 3. Results and Discussions

Cellulose acetate (CA) is a resorbable, non-toxic, environmentally benign, and neutral polymer that forms transparent films. It is highly biocompatible and is obtained from a natural polymer called cellulose. Due to its hydrophilic nature, it can be functionalized by different chemical groups to obtain increased biocompatibility [51]. Membranes made from CA exhibit important chemical stability, good mechanical properties, increased hydrophilicity, excellent protein transport capabilities, low protein adsorption, and water affinity [52]. Cellulose can be prepared from plants, algae, wood, and bacteria, so the annual production is estimated to be about 7 × 10^10^ tons per year [53]. Regarding the CA’s mechanical properties, we can name high elastic modulus, and flexural and tensile strength [54]. The polymer can be used in biomedical applications such as wound-healing patches, drug-delivery systems, and separation membranes [55,56]. The activated hydroxyl groups in CA can be modified or replaced with other functional groups through different methods such as hydrolysis, grafting, oxidation etherification, copolymerization, crosslinking reactions, and esterification [57]. Today, CA polymers with different molecular weights ranging between 30,000 g mol^−1^ [58] and 60,000 g mol^−1^ are used [59]. In order to reduce the Mg alloy corrosion rate, coatings based on CA are used. Demir et al. [60] developed a coating system of a laser-structured surface comprised of a primer layer and a polymeric coating used to improve the degradation behavior of Mg alloys. They used CA as a primer and deposited chitosan and carboxymethyl cellulose layers. A very low corrosion rate of 1.15 cm/year and a hydrophilic characteristic of the implant were obtained. Neacsu et al. [34] coated Mg-Ca-Mn-Zr alloys with cellulose acetate using the dipping method. The polymer coating formation was put in evidence based on the surface characterization method and scanning electron microscopy. The potentiodynamic polarization test proved that the CA coating significantly improved the corrosion of the investigated alloy. The authors also conducted in vitro and in vivo studies that showed increased bone regeneration and good cytocompatibility on MC3T3-E1 preosteoblasts. There are few studies in the literature regarding CA coatings applied on Mg-based alloys despite its high potential in orthopedic applications.

### 3.1. Scanning Electron Microscopy Analysis

**Figure 3:**
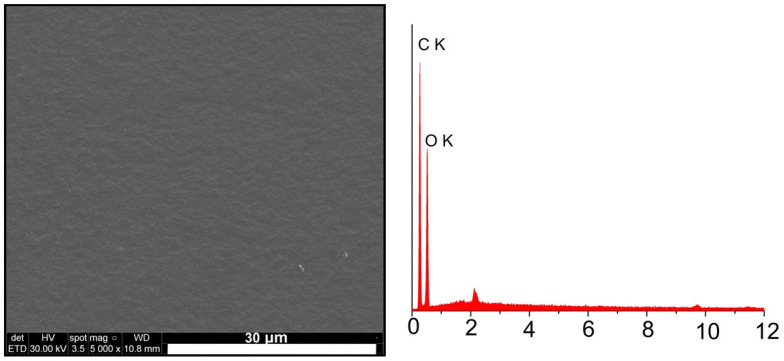
SEM image of the CA sample.

**Figure 4:**
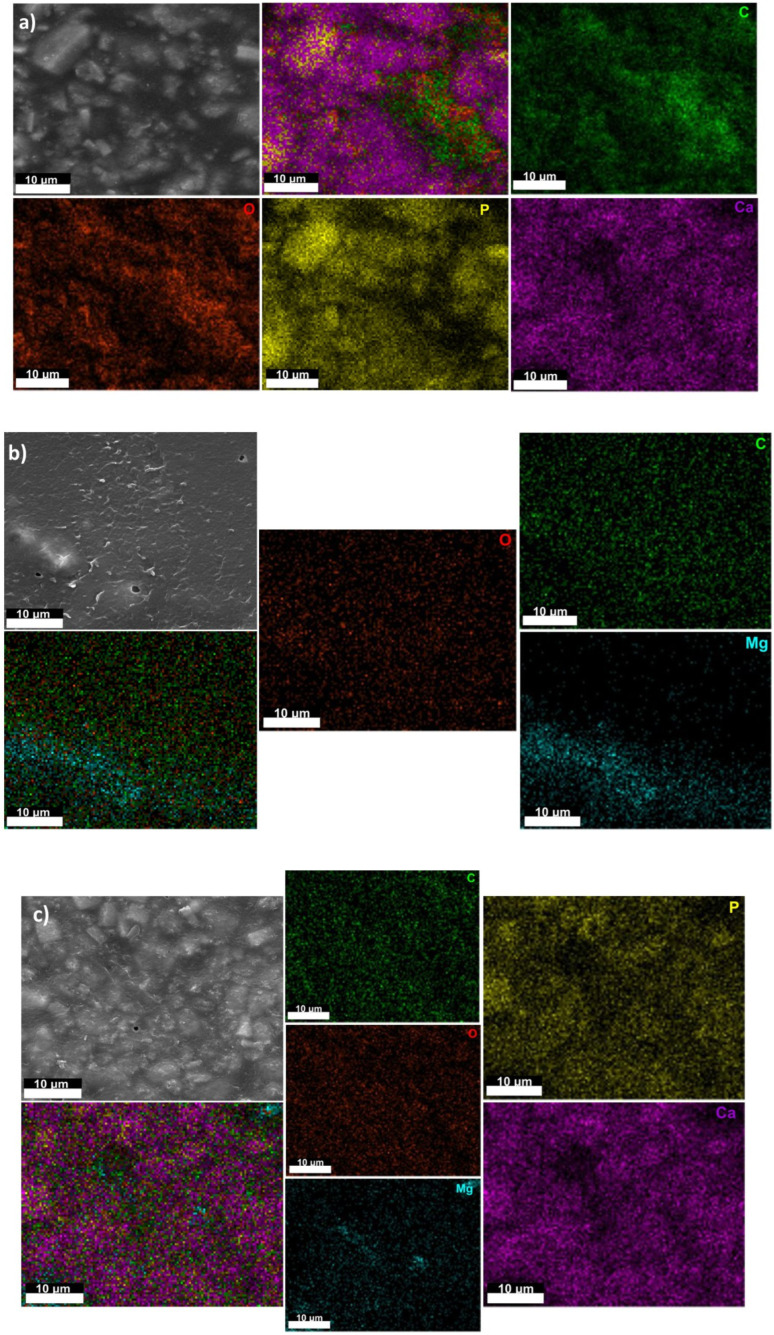
SEM images of the composite sample: (**a**) CA-HAp sample; (**b**) CA-Mg sample; (**c**) CA-HAp-Mg sample.

Figure 3 and Figure 4 show the scanning electron microscopy (SEM) images of the experimental samples, highlighting their surface’s morphological aspects. The SEM image of the CA sample (Figure 3) revealed a smooth surface without polymer formation.

By adding magnesium particles (CA-Mg and CA-HAp-Mg samples), we can notice the appearance of pores of irregular size and distribution in areas of different densities. Pores appear due to the solvent molecules in the experimental sample structure that diffuse outside it during the evaporation process [34,61].

The magnesium particles disperse more uniformly throughout the polymer mass (CA-Mg sample) than the hydroxyapatite particles (CA-HAp sample). Particles of hydroxyapatite appear both in dispersed form or as agglomerates (large crystals).

EDS analysis confirms the presence and distribution of hydroxyapatite powder and magnesium in the structure of the composite material samples. Unlike the sample made from cellulose acetate in whose EDS spectrum (Figure 3) only carbon (C) and oxygen (O) from the polymer structure are identified, in the case of composite samples (Figure 4), the presence of calcium (Ca) and phosphorus (P) from hydroxyapatite and magnesium (Mg) from magnesium particles is observed.

### 3.2. Thermogravimetric Analysis

**Figure 5:**
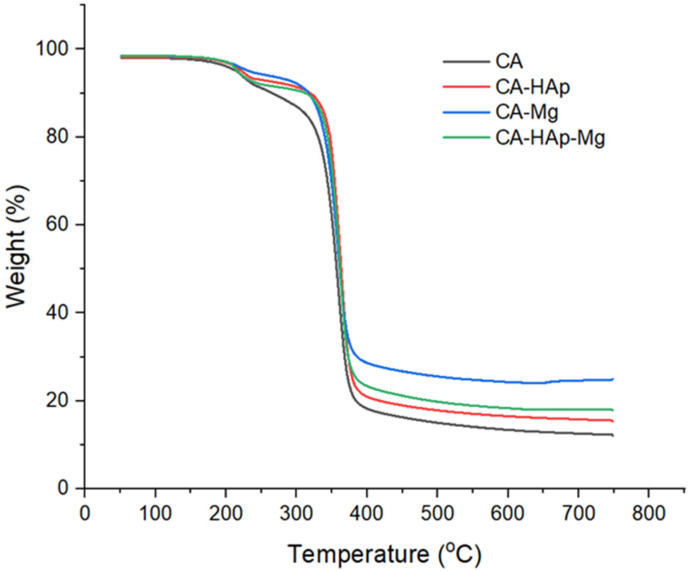
TGA curve of the experimental samples (CA, CA-HAp, CA-Mg, and CA-HAp-Mg).

Thermogravimetric analysis (TGA) is used to study the effect of filler particles on polymer thermostability. Figure 5 illustrates the TGA curves of cellulose acetate, composite coatings with hydroxyapatite particles, magnesium particles, and a mixture of hydroxyapatite and magnesium particles. According to the figure, all samples show a similar profile with two degradation stages. The first stage of degradation is due to the loss of water from the polymer materials, and the second, to the material itself. The lowest thermostability is presented by the cellulose acetate coating, with the thermal resistance increasing with the addition of the filler particles. Despite the similar behavior of the curves, there are significant differences in mass loss; the lowest value is detected in the case of the CA-Mg sample (composite sample with magnesium particles). This fact can be explained by a crosslinking effect that occurs between the magnesium atoms and the non-participating electrons from the oxygen atoms in the polymer component, with these interactions being much stronger than the van der Walls forces that occur between the polymer and the hydroxyapatite particles [62]. It was previously demonstrated that electrostatic interactions that occur in the case of cellulose acetate with magnesium are strong enough and stable in order to increase the thermal resistance of composites, with the atoms implied in these interactions being the oxygen atoms from acetyl groups, with complexant capacity. As a consequence, a crosslinking effect that stabilizes the entire matrix occurs [63,64]. Moreover, the HAp particles form agglomerates, reducing the volume of interaction between the filler and the polymer. The sample CA-HAp-Mg that contains both types of particles (hydroxyapatite and magnesium) has an intermediate behavior, with the thermostability being given by the competition between the two mechanisms of interaction formation.

### 3.3. Fourier Transform Infrared Spectroscopy Analysis

**Figure 6:**
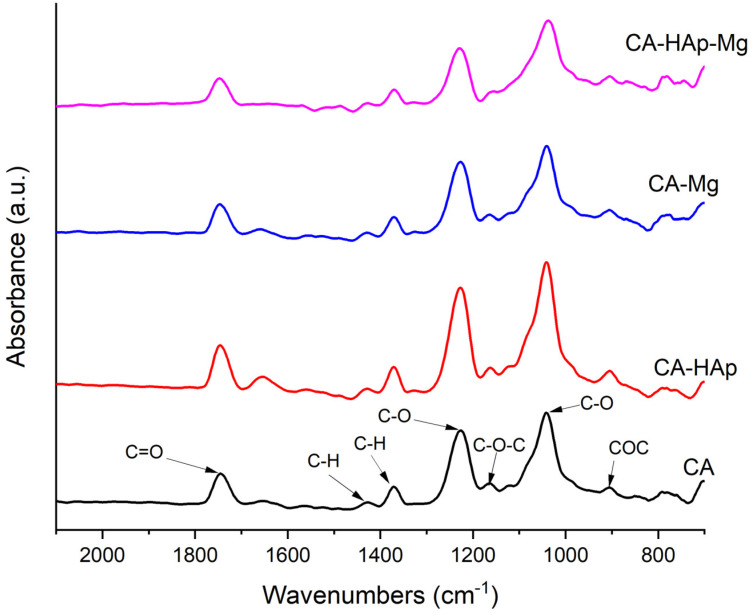
FTIR spectra of the experimental samples (CA, CA-HAp, CA-Mg, and CA-HAp-Mg). The spectra of the composite samples (CA-Mg, Ca-HAp, and CA-HAp-Mg samples) are almost similar to the spectrum of cellulose acetate. The band corresponding to the C-O bond from 1040 cm^−1^ shifts to the right in the case of composite samples (CA-Hap and CA-HAp-Mg) due to the influence of the PO_4_^3−^ groups from hydroxyapatite, which appear in the same region of the spectrum. In the FTIR spectra of the composite samples, there are no two distinct bands for C-O and P-O bond vibrations, due to the small number of inorganic particles contained in and overlapping the vibration bands characteristic of hydroxyapatite in the range of 600–1100 cm^−1^ with the corresponding polymer bands.

Fourier transform infrared (FTIR) spectroscopy is used to identify the specific functional groups or chemical bonds found in the experimental samples (Figure 6), highlighting slight differences between the cellulose acetate sample spectrum and the spectra of the composite coatings’ samples. The spectra exhibit the presence of characteristic bands for cellulose acetate at ~1745 cm^−1^ (υ_C = O_) assigned to the stretching of ester carbonyl (C=O) from the acetyl group, at ~1230 cm^−1^ (υ_C-O_) attributed to the C-O stretching mode from the acetyl group, and at ~1370 cm^−1^ and ~1430 cm^−1^ (δ_C-H_) bands due to the bending vibration of C-H in the -CH_3_ (acetyl group) and -CH_2_ (pyranose ring) groups. Furthermore, at 1040 cm^−1^, a band assigned to the stretching vibration of C-O from pyranose rings appears. Bands at ~1163 cm^−1^ are assigned to COC antisymmetric bridge stretching.

### 3.4. RAMAN Spectroscopy

**Figure 7:**
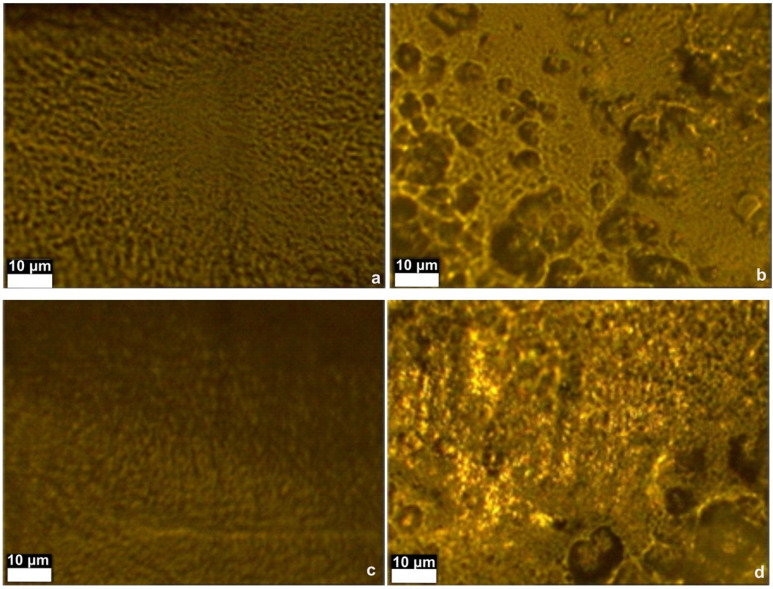
Images (50×) obtained for (**a**) CA sample, (**b**) CA-HAp sample, (**c**) CA-Mg sample, and (**d**) CA-HAp-Mg sample.

In Figure 7, the CA and CA-Mg samples have similar morphologies. This indicates that Mg is uniformly distributed throughout the coatings, in direct correlation with the aspects highlighted by scanning electron microscopy. In contrast, adding HAp particles leads to segregated domains, showing a high degree of conglomeration. This fact may be related to the HAp crystallization and segregation from the solution. The CA-HAp-Mg sample shows some degree of segregation, although the chemistry may be different due to the presence of Mg.

**Figure 8:**
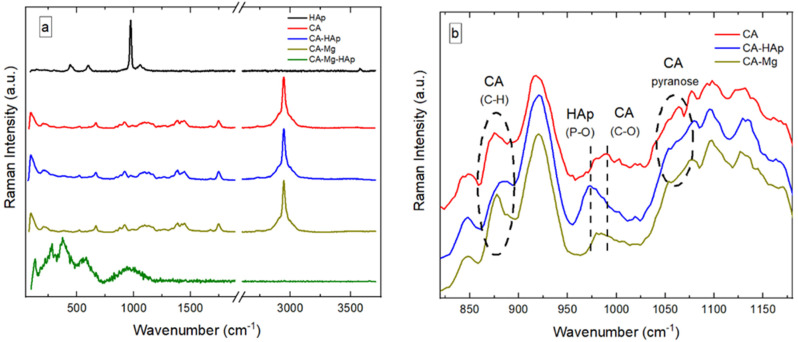
Raman spectra of (**a**) CA and composite samples in 800–1200 cm^−1^ domain; (**b**) CA and composite samples.

The Raman spectrum of hydroxyapatite (HAp) (Figure 8a) presents the main features associated with PO_4_^3−^ and OH^−^ ions [65]. PO_4_^3−^ is characterized by a symmetric stretching (P–O) mode at 973 cm^−1^, bending (O–P–O) mode at 430–450 cm^−1^, anti-symmetric stretching (P–O) mode at 1020–1080 cm^−1^, and bending (O–P–O) mode at 585–610 cm^−1^. The presence of OH^−^ causes the stretching mode at 3600 cm^−1^, vibrational band at 630 cm^−1^, and translational mode at 340 cm^−1^. The contribution from OH- to the Raman spectrum of HAp is weak. Compared to other HAp, the symmetric stretching of the (P–O) mode is shifted to a higher value (i.e., from 960 cm^−1^ to 973 cm^−1^).

In Figure 8a, the Raman spectrum of CA is associated with the vibration bands at 2945 cm^−1^ and 1129 cm^−1^ [66]. They are attributed to C-H stretching and asymmetric stretching vibrations of the C-O-C glycosidic linkage, respectively. In addition, the contribution from the pyranose ring is observed at 1081 cm^−1^. The vibration band associated with the C-OH bonds is observed at 1272 cm^−1^. The characteristic Raman signals for the acetyl group can be observed at 1744, 1443, and 1390 cm^−1^, corresponding to the vibration of the carbonyl group (C=O) and asymmetric and symmetric vibrations of the C-H bond present in the acetyl groups. The vibration bands at 986, 914, 842, and 667 cm^−1^ are associated with C-O, C-H, O-H, and C-OH bonds, respectively.

Overall, adding HAp and Mg to CA leads to subtle transformations in the corresponding vibration bands. As shown in Figure 8b, the addition of HAp to CA results in a weakening of the C-H bond and some vibration associated with the pyranose ring. Additionally, the symmetric stretching (P–O) mode from PO43− in HAp is exhibited at 973 cm^−1^. These features suggest that HAp is crystallized and segregated CA. Similar results are found for samples containing Mg and CA, with the difference that the contribution to the vibration of the C-H bond is enhanced, whereas some vibration associated with the pyranose ring is suppressed. However, the addition of both Mg and HAp to the CA coatings leads to dramatic changes in the Raman spectrum (CA-Mg-HAp sample, in Figure 8a). The overall features exhibited by CA are suppressed above 1100 cm^−1^, indicating that the back-bone chemistry of the membrane is drastically changed. The absence of the O-H stretching vibration of pure water in the region of 3600–3800 cm^−1^ indicates that the membrane is dehydrated. The main Raman vibration bands of the CA-Mg-HAp membrane exhibited at 127, 275, and 380 cm^−1^ are sharp. The Raman bands at 573 and 952 cm^−1^ are weak and broad. This makes it difficult to quantify the chemistry of the specimen. According to the literature data, the Raman bands between 100 and 210 cm^−1^ are lattice (phonon) vibrations with strong intensity. This might be related to the precipitation of nano-Mg(OH)2 or MgO phases with reduced crystal symmetry. However, C-C aliphatic chains have strong vibrations in the range of 250–400 cm^−1^. This accounts for the Raman peaks at 275 and 380 cm^−1^. The weak vibrations at 573 cm^−1^ may be related to C-(I, Cl, or Br) groups (i.e., with vibrations between 490 and 790 cm^−1^). The broad band at 952 cm^−1^ may be associated with the C-O-C group (i.e., vibrations at 800–950 cm^−1^) and/or the carboxyl acid dimer (i.e., vibrations at 910–960 cm^−1^).

### 3.5. Degradation and Swelling Studies

**Figure 9:**
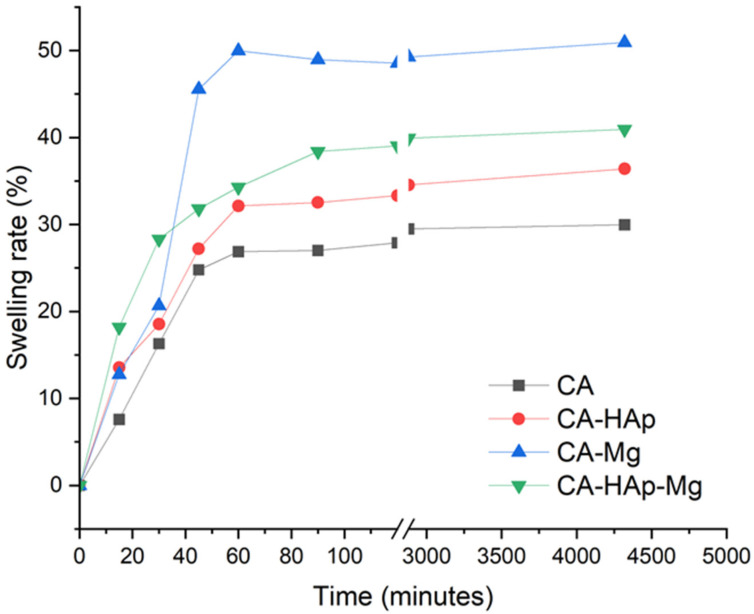
Swelling of the experimental samples over a period of 72 h in PBS solution.

The swelling rate and degradation behavior of the experimental samples are shown in Figure 9. The swelling rate is an important indicator regarding the absorption and retention capacity of liquids by a polymer matrix. It is observed that with the addition of hydroxyapatite and magnesium particles, the swelling capacity increases. This aspect is due to the porosity of the composite samples that allow the capture of more water molecules. This aspect leads to a more intense degradation process.

In addition, measuring the contact angle to determine the hydrophilic or hydrophobic characteristics of the samples’ surface highlights their hydrophilicity. Moreover, with the addition of hydroxyapatite and magnesium particles in the polymer matrix, the hydrophilic characteristic increases and, consequently, the swelling capacity of the samples. The hydrophilic character favors a better interaction between samples and the PBS solution, which is mostly water.

From Figure 9, it can be seen that in the first 45 min, the swelling rate increases rapidly for all samples but is more pronounced for those with magnesium particles (CA-Mg and CA-HAp-Mg). The samples reach a steady state after 90 min, with a swelling of about 48% for the CA-Mg sample, 38% for the CA-HAp-Mg sample, and 32% for the CA-HAp sample.

**Figure 10:**
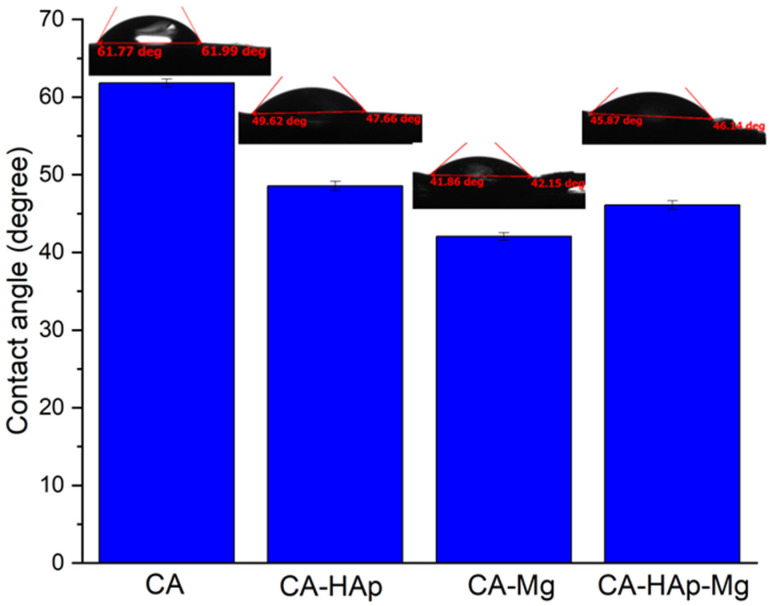
Contact angle values of the experimental samples.

The wetting of the surface, quantified by the contact angle value measurements (Table 4), represents an important factor in the functionality and biocompatibility of implantable devices [1,3]. Thus, a small contact angle will improve cell adhesion, while a hydrophobic surface (contact angle > 90°) could affect cell adhesion, leading to rejection of the implantable material. The lowest contact angle values are obtained for samples containing magnesium particles (CA-HAp-Mg and CA-Mg), which proves that by adding them, hydrophilic surfaces favorable for biological integration are obtained (Figure 10). It is well known that when a liquid droplet comes into contact with a mixture of different particles, such as in our case (HAp and Mg particles), a complex contact angle behaviour can be observed [67]. As an analytical tool, the Cassie–Baxter theory can be used. Mundozah et al. [67] adapted this model by considering the average particle diameter. They showed that in the case of a mixture formed from larger particles (i.e., Mg powder) and small particles (i.e., HAp powder), if the larger particle diameter is higher than a critical volume fraction, the surface wettability could change considerably due to the fact that partial coverage of larger particles by the small ones appears. They also stated that for larger particle diameters less than or equal to the critical value, the full surface coverage of larger particles occurs, and the mixture receives the surface characteristics of small particles. In our study, it can be noticed from Figure 10 that in the case of CA-HAp-Mg, the contact angle is almost equal to that obtained for CA-HAp. We can conclude that due to the fact that HAp small particles can easily form agglomerates, as evidenced from SEM investigations, the HAp particles cover in a large amount the surface of Mg particles. This fact determines an increase in the surface contact angle in comparison with that obtained for the CA-Mg sample.

### 3.6. Degradation Behavior

**Figure 11:**
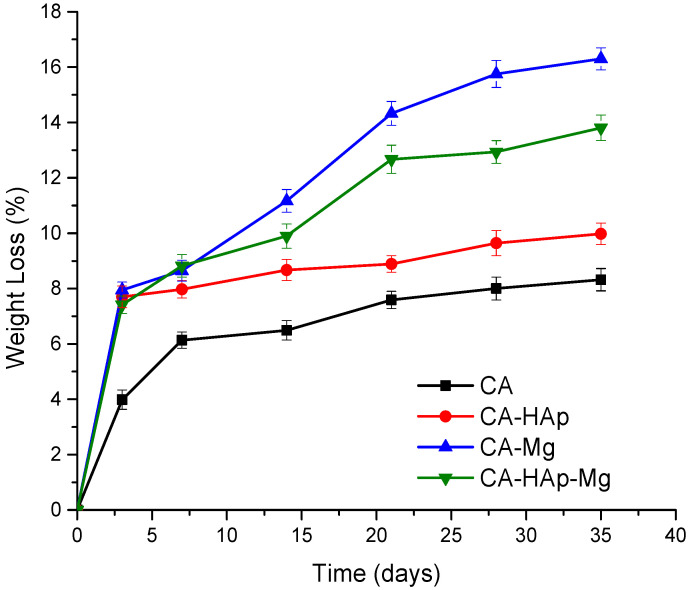
Degradation profile of the experimental samples calculated by weight loss over a period of 35 days of immersion in PBS solution.

The degradation behavior of the experimental samples is evaluated by measuring the weight loss. The evolution of weight loss evaluated at 3, 7, 14, 21, 28, and 35 days of immersion in PBS is shown in Figure 11. The degradation process takes place faster in the first 3 days of immersion in all the samples. During the following periods, the CA and CA-HAp samples show lower degradation rates, with the increase being smaller and constant. The samples containing magnesium particles show a more intense degradation behavior, recording a weight loss of 15.99% for the CA-Mg sample and of 13.21% for the CA-HAp-Mg, double that of the first 3 days of immersion.

Comparing samples containing magnesium particles, the lower weight loss value is obtained for the sample containing a mixture of hydroxyapatite and magnesium particles (CA-HAp-Mg), which highlights that the degradation process given by the magnesium particles is repelled by the hydroxyapatite presence.

The composite samples that are the subject of this study are obtained to be used as coatings for magnesium alloys. Magnesium and its alloys are known to be promising as orthopedic materials, especially as bone fixation devices or implants.

### 3.7. MTT Assay

**Figure 12:**
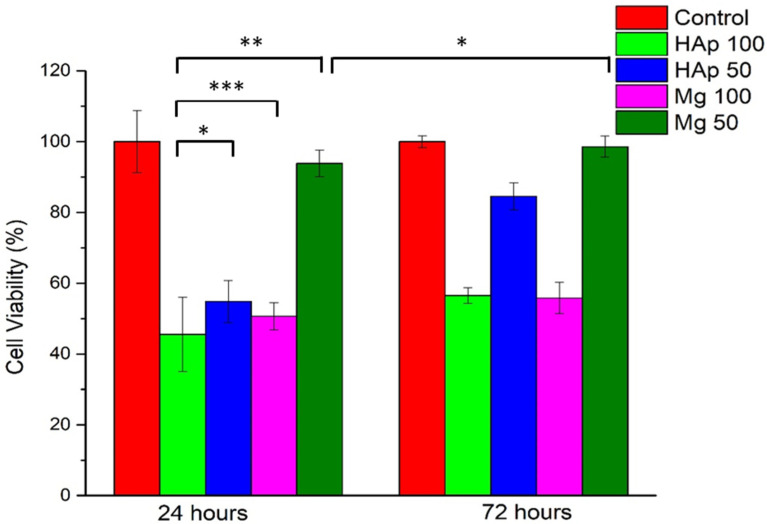
The cell viability, measured by the MTT assay for indirect contact (extracts), where 100 represents 100% extract and 50, 50% extract. Values were expressed as the mean ± SD from three independent experiments (*n* = 3). Each value represents the mean ± standard error mean (*n* = 3). * *p* < 0.01, ** *p* < 0.001, and *** *p* < 0.0001 versus control (analyzed by means of two-way ANOVA with Tukey’s post hoc analysis).

**Figure 13:**
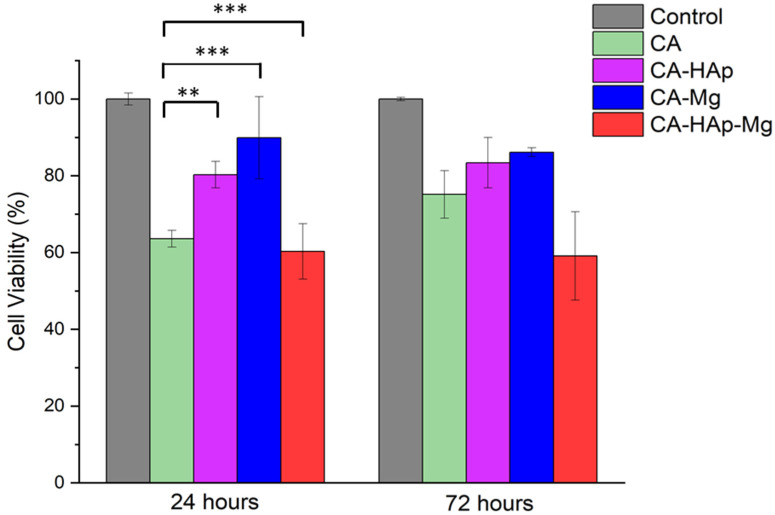
The cell viability, measured by the MTT assay for direct contact: ** *p* < 0.001, and *** *p* < 0.0001 (analyzed by means of two-way ANOVA with Tukey’s post hoc analysis).

MTT results are shown in Figure 12 and Figure 13. Considering the tested inorganic particles used as fillers (Figure 12), it is clear that 100% extract is cytotoxic, with the values obtained at 24 h being 45.54% for HAp and 50.65% for Mg, while at 72 h, it is found to be 56.58% for HAp and 55.86% for Mg. In the case of HAp, the cytotoxic behavior is also maintained for 50% extract at 24 h (54% viability), but an interesting phenomenon is observed at 72 h, when the cell viability increases significantly at 84%, suggesting a slightly initial toxic effect. Regarding Mg particles, it can be stated that this material is non-cytotoxic at a concentration of 50% extract at both contact times.

For the experimental samples tested in direct contact with cells, the results are represented in Figure 13. For CA (cellulose acetate) at 24 h, the cell viability is 63% and, at 72 h, increases to 75%. These values can be explained by a slightly acidic characteristic, mentioned in the methods section, which leads to the acidification of the culture medium. An important observation is related to the fact that by adding HAp and Mg, the cell viability increases to 70% (24 h) and 80% (72 h) for CA-HAp, and 89% (24 h) and 86% (72 h) for CA-Mg. These results are directly correlated to the results obtained for HAp and Mg extracts, with Mg being non-cytotoxic at 50% extract (93% viability at 24 h and 99% viability at 72 h, Figure 12). Mixing CA with both HAp and Mg decreases cell viability, and several tests are necessary to establish the amount in which they must be combined.

**Figure 14:**
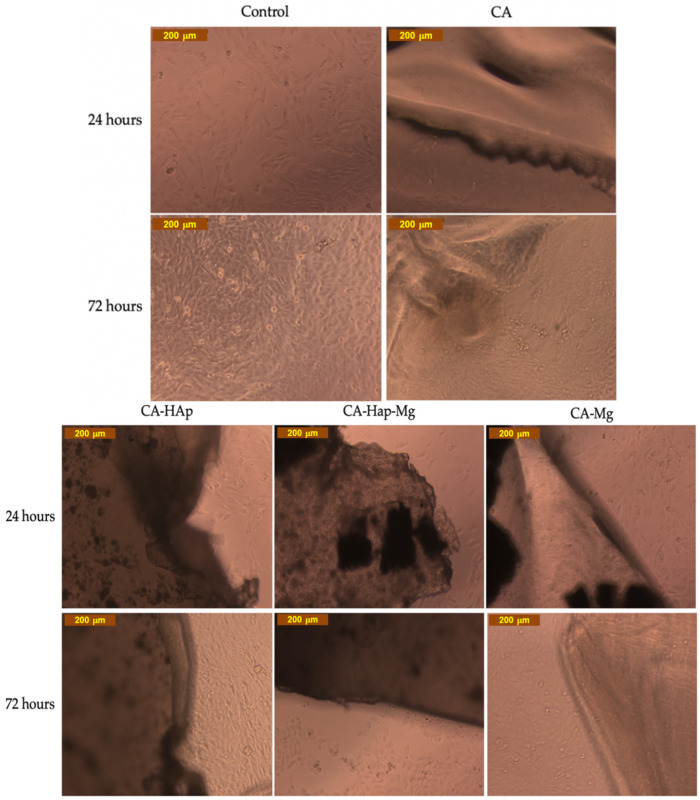
Optical microscopy images of the MG-63 cell line in direct contact (24 and 72 h) with the experimental samples. Image obtained with Inverted Phase-Contrast Microscope, Leica, Germany (10× objective).

Regarding the morphology and density of cells, Figure 14 shows that in the case of cells incubated with a CA-HAp sample, these parameters are very similar, which indicates a non-cytotoxic effect, a result that is in contrast with MTT, in which cell viability values of 70% and 80% (72 h) are obtained. The explanation is that when the material aliquot is removed before adding the MTT solution, some cells that adhered to the material are also removed. This is a promising result, indicating the capacity of the sample to sustain cell adhesions. For the other experimental samples, CA, CA-HAp-Mg, and CA-Mg, the morphology and density of cells confirm the MTT results.

### 3.8. Calcein-AM Cell Viability Assay

**Figure 15:**
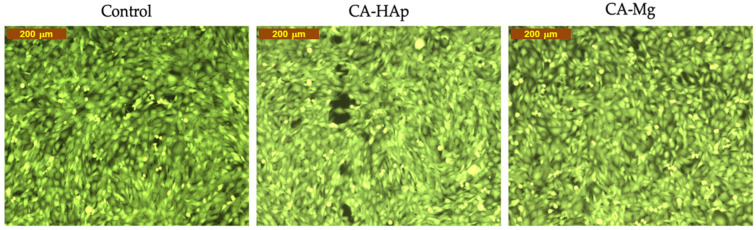
Fluorescent microscopy images for CA-HAp and CA-Mg in contact with cell cultures for 72 h (dye Calcein AM). Image obtained with Inverted Phase-Contrast Microscope, Leica, Germany (10× objective).

Calcein-AM is the most representative dye from the live cell non- or low-fluorescent-dyes class. These dyes easily penetrate cells’ plasma membranes, where they are converted into impermeable fluorescent products; in the case of Calcein-AM, the reaction is possible due to the intracellular esterases, resulting in a green fluorescent product [68]. MTT assays highlight that the viability of cells incubated with CA-HAp is 80% and that with Ca-Mg is 86% after 72 h of direct contact; in both cases, the current assay also confirms the values. The Calcein AM Cell Viability Assay is generally used to support the MTT data, by imaging the viable cells, and can give some indications about changes in cells’ morphology. In Figure 15, comparing the images corresponding to the CA-HAp and CA-Mg materials, the existence of gaps in the cellular layer can be observed for CA-Hap, suggesting lower cell viability for this composition.

## 4. Conclusions

The major concerns regarding Mg-based biomaterials are their uneven and rapid degradation. In order to counteract this disadvantage, this study aimed to obtain composite polymer coatings that have stability in physiological environments and present a good osteoblast response in terms of cell adhesion and viability. The study demonstrated that coatings based on cellulose acetate and hydroxyapatite and/or magnesium particles could be obtained by the solvent evaporation method. TGA analyses confirmed the stability of the samples up to 200 °C and highlighted a mass loss between 5 and 9% in the temperature range of 25–250 °C, attributed to the presence of water in the material structure. After 250 °C, the coatings showed significant weight loss due to the polymer degradation. The addition of hydroxyapatite in the cellulose acetate polymer matrix influenced the composite samples’ morphology. Scanning electron microscopy highlighted the formation of hydroxyapatite crystals in the material’s structure (CA-HAp and CA-HAp-Mg) because of the poor dispersion of the inorganic filler in the polymer solution. In the case of CA-HAp and CA-Mg composite samples, homogeneous structures and cell viability values above 80% were obtained. The analyses carried out on the experimental composite samples highlighted the positive effect of magnesium and hydroxyapatite particles when they are used alone. It is not recommended to use both types of particles (hydroxyapatite and magnesium) as hybrid filling. In future studies, we will use only an inorganic filler to obtain CA-based composite coatings on magnesium alloys because these composite coatings showed better results from the in vitro testing point of view for future potential orthopedic biodegradable implants for trauma.

## Figures and Tables

**Table 1:** Characteristics and effects of synthetic polymer coatings on Mg-based alloys.

Polymer	Polymer Properties	Coating	Mg-Based Substrate	Coating Technology	Remarks	Ref.
Polylactic Acid (PLA)	Biodegradable, Young’s modulus of about 3 GPa, ultimate tensile strength (UTS) of 50–70 MPa, fracture toughness of about 2.5 kJ/m^2^, biocompatible, degrades through hydrolysis	Poly(L-lactic acid) (PLLA)	Pure Mg rods	Dip-coating	PLLA coatings with porous structures and dense surfaces were obtained. The corrosion resistance was improved, and the lower pH of the coating sustained the emission of Mg^2+^ ions	[30]
		PLA	Mg-9Al-1Zn (AZ91)	Spin-coating	PLA coating improved the corrosion behavior of the Mg-based alloy	[29]
Poly(Lactide-Co-Glycolic) Acid (PLGA)	High biocompatibility, already approved for use in human clinical trials, biodegradability, exhibits controllable degradation properties	PLGA	Mg-6Zn	Dip-coating	The degradation rate was reduced by a high amount	[31]
Polycapro lactone (PCL)	PCL is Food and Drug Administration (FDA)-approved. Biocompatibiliy, biodegradability, nontoxicity, high strain failure rate	PCL	Mg-based scaffolds produced through powder metallurgy route	Dip-coating	The degradation rate was reduced	[33]
		PCL	Mg-6%Zn-10%Ca_3_(PO_4_)_2_ scaffolds	Surface sprayed and solidified at 50 °C for 5 min	Corrosion activity, surface morphology, and cytotoxicity were improved	[32]
Cellulose acetate (CA)	It increases corrosion resistance. CA is a thermoplastic material, and its main source is cellulose that is a natural polymer. CA is not manufactured by polymerization of any monomer	CA	Mg-Ca-Mn-Zr	Dip-coating	The coating increased the corrosion resistance. The material exhibited good cytocompatibility, promoting cell adhesion, viability, proliferation, and osteogenic differentiation	[34]

**Table 2:** Different types of composite coatings on Mg-based alloys.

Composite Coating	Type of Composite Coating	Mg-Based Substrate	Coating Technology	Remarks	Ref.
Polylactic Acid (PLA)/Brushite	Synthetic polymeric/inorganic non-metallic ceramic based on phosphate	Mg-Nd-Zn-Zr	Composite coating with an inner layer of PLA and an outer layer of brushite prepared through chemical deposition	The biocorrosion resistance and biocompatibility were increased. The developed materials can be used as drug-delivery systems.	[48]
Micro-arc oxidation (MAO) + Poly(Lactide-Co-Glycolic) acid (PLGA) composite	Metallic/Synthetic polymeric	Mg-4Zn-0.6Zr-0.4Sr	Micro-arc oxidation	The MAO+PLGA coating increased the corrosion and stress corrosion cracking resistance of the Mg-based alloy. The high mechanical stability of the coated alloy was put in evidence.	[49]
Chitosan (CS)/heparinized graphene oxide (HGO) composite film	Natural polymeric/inorganic non-metallic based on graphene oxide	Mg-3Al-1Zn (AZ31B)	Layer-by-layer self-assembly method (LBL)	The CS/HGO composite coating degraded slowly in simulated body fluid (SBF) solution and offered the Mg-based alloy high corrosion protection. The multilayer film promoted the proliferation and adhesion of the endothelial cells and played an essential role in the reduction in the hemolysis rate.	[45]
Highly dense Hydroxyapatite (Hap)/Chitosan (CS) film	Inorganic non-metallic ceramic based on phosphate/natural polymeric	Mg-3Al-1Zn -Mn (AZ31)	Aerosol deposition	The aerosol deposition is a method used to manufacture high-density ceramic-polymer composite coatings. The coated samples exhibited a high adhesion phenomenon and a reduced corrosion rate. The Mg-based alloy biocompatibility was highly improved.	[46]
Bi-layered functional coatings consisting of an inner silane–TiO_2_ coating and an outer polymeric layer made from collagen (Col) or chitosan (CS)	Metallic/Natural polymeric	Mg-3Al-1Zn (AZ31), Mg-4Zn-2RE-0.7Zr (ZE41)	Sol–gel deposition and thermal conditioning	The coating has a beneficial effect against corrosion. High biocompatibility was put in evidence due to natural polymers’ existence. The bi-layered coating prevents the hydrogen from being released.	[47]
Polypyrrole (Ppy)/Gelatin (Gel)	Synthetic polymeric/natural polymeric	Mg-3Al-1Zn (AZ31)	Anodization and electrodeposition	A composite coating, which contains an anodization film and a Ppy/Gel layer, increases the corrosion resistance and reduces the H_2_ elimination	[42]

**Table 3:** Composition of the elaborated samples.

Sample	Composition
CA	Cellulose acetate
CA-HAp	Cellulose acetate + 5% hydroxyapatite
CA-Mg	Cellulose acetate + 5% Mg particles
CA-HAp-Mg	Cellulose acetate + 5% hydroxyapatite + 5% Mg particles

**Table 4:** Contact angle values.

Samples	Contact Angle (Degree)
CA	61.80 ± 0.53
CA-HAp	48.54 ± 0.62
CA-Mg	42.05 ± 0.49
CA-HAp-Mg	46.09 ± 0.55

## Data Availability

Not applicable.
